# Influence of Football on Physiological Cardiac Indexes in Professional and Young Athletes

**DOI:** 10.3389/fphys.2018.00153

**Published:** 2018-02-28

**Authors:** Cristian V. Francavilla, Francesco Sessa, Monica Salerno, Giuseppe D. Albano, Ines Villano, Giovanni Messina, Fabio Triolo, Lorenzo Todaro, Maria Ruberto, Gabriella Marsala, Orazio Cascio, Maria P. Mollica, Vincenzo Monda, Giuseppe Cibelli, Anna Valenzano, Christian Zammit, Marcellino Monda, Antonietta Messina

**Affiliations:** ^1^Universita degli Studi di Enna “Kore”, Enna, Italy; ^2^Department of Clinical and Experimental Medicine, University of Foggia, Foggia, Italy; ^3^Department of Experimental Medicine, Section of Human Physiology and Unit of Dietetics and Sports Medicine, Università degli Studi della Campania “Luigi Vanvitelli”, Naples, Italy; ^4^Department of Surgery and Oncology - UOC di Cardiochirurgia - “P. Giaccone” Hospital, Palermo, Italy; ^5^Unione Sportiva Città di Palermo, Palermo, Italy; ^6^CRD Center, Santa Maria del Pozzo, Somma Vesuviana, Italy; ^7^Struttura Complessa di Farmacia, Azienda Ospedaliero-Universitaria, Foggia, Italy; ^8^Department of Anatomy, University of Catania, Catania, Italy; ^9^Department of Anatomy, University of Malta, Msida, Malta

**Keywords:** professional football player, echocardiographic parameters, heart adaptation to exercise, left ventricle dimensions, athlete's heart

## Abstract

**Background:** After long-term intensive training, considerable morphological and functional heart changes occur in professional athletes. Such changes arise progressively and regress upon interruption of the physical activity. Morphological and functional alterations on heart are known as “Athlete's heart” condition.

**Objective:** This study aims to compare echocardiographic parameters in two different groups of professional athletes. Furthermore, a prospective study is performed analyzing the echocardiographic changes occurring in 12 professional players in 3 years of follow-up.

**Materials and Methods:** 78 football players were examined from July 2011 to May 2016 (40 enrolled in Group A and 38 in Group B). Twelve players of GROUP A were followed for 3 consecutive seasons. The general clinical examination, the cardiopulmonary evaluation, the ECG, the ergometer stress test, the spirometric examination and the standard cardiac eco color doppler test were recorded.

**Results:** Left ventricle dimensions, left atrium dimensions, and interventricular septum dimensions were higher in A players than in B players. Moreover, following up 12 players for 3 years, a statistically significant increase of such values was observed.

**Discussion:** In A players, higher dimensions of the left chambers and the interventricular septum were observed, compared to B players. No statistically significant difference was found regarding the ejection fraction. The 3 years follow-up showed a statistically significant increase of both left chambers and interventricular septum dimensions, particularly in the second and third year.

**Conclusions:** These findings demonstrated that A players have higher echocardiographic parameters respect to B players. The results of this study support the scientific theory that long-term intensive training influences heart function, inducing “athlete's heart” with morphological adaptations. No significant echocardiographic variation within the examined sample was observed for different roles (goalkeeper, defender, midfielder, or attacker) or skills of individual players.

## Introduction

Physical activity is an essential tool in order to prevent cardiovascular, musculoskeletal and metabolic diseases. Sedentary lifestyle is still widespread among both youths and adults, especially in the western countries (Francavilla et al., 2007).

Long-term intensive training program leads to several morphological and functional cardiovascular changes that occur progressively, and regress upon interruption of the physical activity (Atchley and Douglas, 2007; Zeppilli, 2008; D'Andrea et al., 2009; Neri et al., 2009a).

“Athlete's heart” is defined as the adaptation of the heart to long-term intensive physical activity, and is characterized by symmetric and harmonic increase of intracavitary diameters and wall thickness (Fagard, 2003; Turillazzi et al., 2010).

Such adaptation is strictly dependent on a wide spectrum of factors: (1) genotype (hereditary factors) (Sessa et al., 2011); (2) age of onset of physical activity; (3) type and intensity of training program (Lavie et al., 2001).

Two different intensive physical activity models were initially described, both related to different ventricular hypertrophy models.

Resistance training is characterized by isotonic-dynamic muscular activity and requires aerobic energy expenditure. It gradually leads to a decrease in both systemic arterial resistance and venous return, and a telediastolic left ventricle volume increase, leading to an increase in systolic output. Resistance athletes (swimming, long distance running, cycling etc…) show an increase in all the intracavitary dimensions, and also in wall thickness. These features lead to eccentric hypertrophy due to the volume overload, with a proportional increase in myocardial mass (Neri et al., 2009b; Lamotte et al., 2010; Forlano et al., 2011).

Power training is characterized by static muscular activity and requires anaerobic energy expenditure. It leads to a consistent increase in myocardial mass, due to the increase in wall thickness. The increase in intracavitary diameters is insignificant, and therefore results in a concentric ventricular hypertrophy. These changes are frequently found in athletes practicing weightlifting, short distance running, etc….

Such adaptations are involved in isometric muscular work. They are characterized by a pressure response to prolonged muscular tensions, with no increase in regional perfusion. The increase in peripheral resistance leads to a substantial raise in left ventricular afterload during exercise, resulting in left ventricle pressure overload (Messina et al., 2016; Chieffi et al., 2017; Maté-Muñoz et al., 2017).

The above-mentioned model, characterized by two different training mechanisms, is no longer reputed accurate. Recent studies suggest a unique heart adaptation model, characterized by a ventricular hypertrophy correlated to body size and exercise intensity, not to the nature of the physical activity (Pelliccia et al., 1991, 1999; Spirito et al., 1994; Avola et al., 2004; Naylor et al., 2008; Zeppilli et al., 2008; Cianci et al., 2016).

The physiological demands of soccer are complex and they are related to the nature of the exercise pattern (Morgans et al., 2014). According to the Mitchell et al. classification (Mitchell et al., 2005), football players usually perform a physical activity that includes both a dynamic and a static component. The requirement for frequent changes in both the speed of movement (e.g., walking, jogging, high intensity running, and sprinting) and direction, makes the activity profile intermittent. In fact, football, like most other sports, is characterized by both aerobic and anaerobic energy expenditure, with an overload in both left ventricular volume and pressure. In athletes who practice this sport, there is an increase in both intracavitary diameters and walls thickness. This results in an intermediate ventricular hypertrophy model, between resistance exercise and power exercise (Bangsbo et al., 2006; Bramanti et al., 2012; Mielgo-Ayuso et al., 2015; Precenzano et al., 2016a; Zurutuza et al., 2017).

After a long-term intensive exercise, professional athletes undergo substantial morphological and functional cardiovascular changes, which arise progressively and regress upon reduction of physical activity. During the last decades, sports culture has changed consistently. Professional athletes follow rigorous training programs with daily practice sessions, leading to a wide range of cardiovascular and musculoskeletal adaptation mechanisms. These changes are related to other factors, such as genotype, age, and age onset of physical activity.

One of the most important adaptations is named “Athlete's heart,” involving hearth changes. Several studies showed how correct interpretation of electrocardiography could lead to a correct interpretation of the features present in the “Athlete's heart” (Braschi et al., 2009, 2011, 2012; Neri et al., 2013; Vyshka and Vacchiano, 2014; Francavilla et al., 2016).

This study aims to compare echocardiographic parameters in two different groups of professional athletes. Furthermore, a prospective study is performed analyzing the echocardiographic changes occurring in 12 professional players in 3 years of follow-up.

## Materials and methods

Seventy eight professional football players were enrolled subdivided into two groups. Group A is composed of 40 “Serie A” players (“Serie A” is a professional league competition for football clubs located at the top of the Italian football league system); Group B is constituted by 38 “Primavera” players of the respective team (“Primavera” is named the Italian youth league, under 19 y.o. football players). All evaluations were recorded between July 2011 and May 2016. The principal biological parameters for each group are summarized in Table 1. All athletes were evaluated at the beginning of the regular season; 12 players of GROUP A were followed for 3 consecutive seasons. No lifestyle changes were reported. A complete cardiovascular examination was performed on all athletes. The general clinical examination, the cardiopulmonary evaluation, the ECG, the ergometer stress test, the spirometric examination and the standard cardiac eco color doppler test were recorded.

**Table 1:** The principal biological parameters for each group.

**Parameters**	**Group A**	**Group B**
Mean age	26.6 ± 3.1	18.3 ± 0.6
Mean height (m)	1.85 ± 0.08	1.83 ± 0.04
Mean weight (Kg)	78 ± 8.6	75.5 ± 5.2
Mean BMI	22.8 ± 1.13	22.5 ± 0.8
Heart rate range	37–58 bpm	45–64 bpm
Mean blood pressure	70/110	70/110
% SpO_2_	99%	99%

All subjects gave written informed consent in accordance with the latest version of the declaration of Helsinki. The protocol was approved by the Human Ethical Review Committee (University of Palermo).

### Training program in professional football players

All athletes were trained following the typical training protocol for each season. A regular season consists of 11 working months and is preceded by 3 weeks of intensive training characterized by training twice daily. In a typical training week, the players undergo six practice sessions and 1 official game, which usually take place on Sunday. Each player is trained for a total amount of 12–14 h of physical activity per week. Most of the physical activity is performed on the football field, with a once weekly training session taking place in the gym.

### Body composition and anthropometric parameters

For the body weight measurement the Seca 877 Mechanical Scale (Hamburg, Germany) weight scale was used. Body length measurement was performed using SECA 240. Bioimpedance analysis was performed using BIA 101 ASE (Akern) instrumentation. Resistance and reactance were measured in standard condition, at rest and 12 h after the last training session. The subcutaneous tissue thickness was also measured using the Wither formula to estimate the fat mass percentage.

### Echo color doppler evaluation

The echocardiographic evaluation was conducted by an experienced cardiologist using a Philips Ie33 device, withan X-Matrix 2.5 MHz probe. The exam was always conducted by the same operator to eliminate inter-operator variability. In accordance with the American Society of Echocardiography guidelines, both systolic and ventricular diastolic parameters were measured at rest.

Echocardiographic evaluation included measuring of wall thickness and intracavitary diastolic and telediastolic diameters. Cardiac chambers dimensions were calculated in ml/mq. These data were differentiated according to the training mechanism involved.

Both systolic and diastolic interventricular septum thickness, posterior wall thickness, left ventricle diameter, aortic root diameter and left atrium dimensions were calculated. The Devereux formula was used in order to calculate left ventricular mass index (g/m^2^) (Ganau et al., 1992). The Simpson formula was used in order to calculate ejection fraction. Diastolic function was evaluated by calculating E and A waves and deceleration time (TD). In addition, by using tissue doppler, E', A', S', and E/E' ratio were calculated, to estimate left ventricular filling pressures.

### Statistic analysis

For each of the variables related to the analysis, the mean, standard deviation, median, minimum and maximum, were calculated. The left ventricle size, the left atrium, the interventricular septum, and the ejection fraction were compared between Group A and B using the T Student test.

A team variables comparison was performed by using the ANOVA model. If requested, Tukey *post-hoc* test was calculated for multiple comparing.

## Results

All data were expressed as mean and standard deviation values (SD). The assessment of body composition showed that the Group A weighed more than the B group, with a higher active cellular mass and a lower fat mass percentage. Group A players also had a better hydration state. The principal parameters for each group were summarized in Table 1.

In the examined cohort, the diastolic left ventricle size was between 40 and 61 mm, and the left atrium volume was between 21.3 and 40 ml/m^2^. The interventricular septum size was between 7 and 11.9 mm, and showed a directly proportional trend with the athlete's age and professional physical activity duration.

The ejection fraction had values between 60 and 70%. A higher than normal regional diastolic longitudinal functional myocardial pattern was observed in 85% of athletes, and it was characterized by high myocardial pro-diastolic velocities, as well as an increased Em/Am ratio. Em velocity is directly correlated to telediastolic diameter and systolic output under physical effort. Cardiac valves function was also evaluated; no structural valvular changes were observed. In seven athletes a low-grade tricuspid insufficiency was detected, in eight cases a low-grade mitral insufficiency was observed, and in five cases both tricuspid and mitral insufficiency were observed.

**1. Group A vs. B: Left Ventricle Size Comparison**

**Figure 1:**
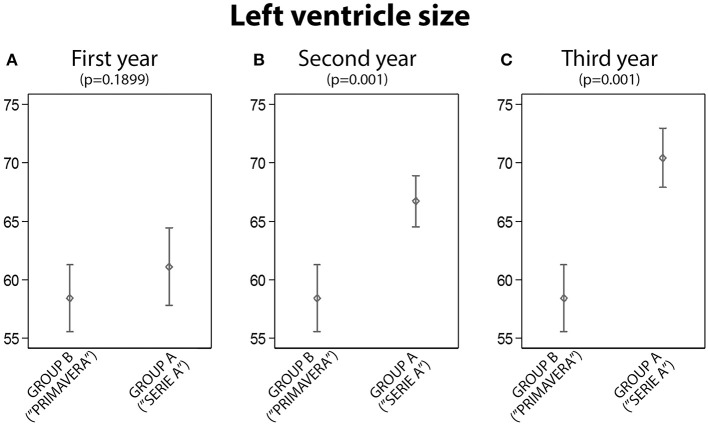
The analysis of left ventricle size between Group A and Group B: statistically significant differences were found in the second and third year (**A**, First year; **B**, Second year; **C**, Third year).

Analyzing the left ventricle size (expressed in ml/mq), no statistical difference was found between the GROUP A and the GROUP B (Figure 1A). A statistically significant difference was found between the two groups after the second year (58.4 vs. 66.7, *p* < 0.001) and after the third year (60.2 vs. 70.4, *p* < 0.001), Figures 1B,C.

**2. Group A vs. B: Left Atrial Size Comparison**

**Figure 2:**
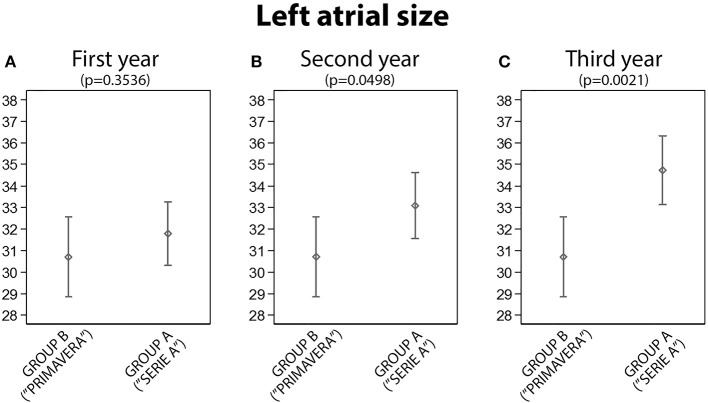
The analysis of left atrial size between Group A and Group B: statistically significant differences were found in the second and third year (**A**, First year; **B**, Second year; **C**, Third year).

Atrial size indexed evaluation showed a statistically significant difference between the two groups after the second and the third year; no significant difference was found after first year (Figures 2A–C).

**3. Group A vs. B: Interventricular Septum Size Comparison**

**Figure 3:**
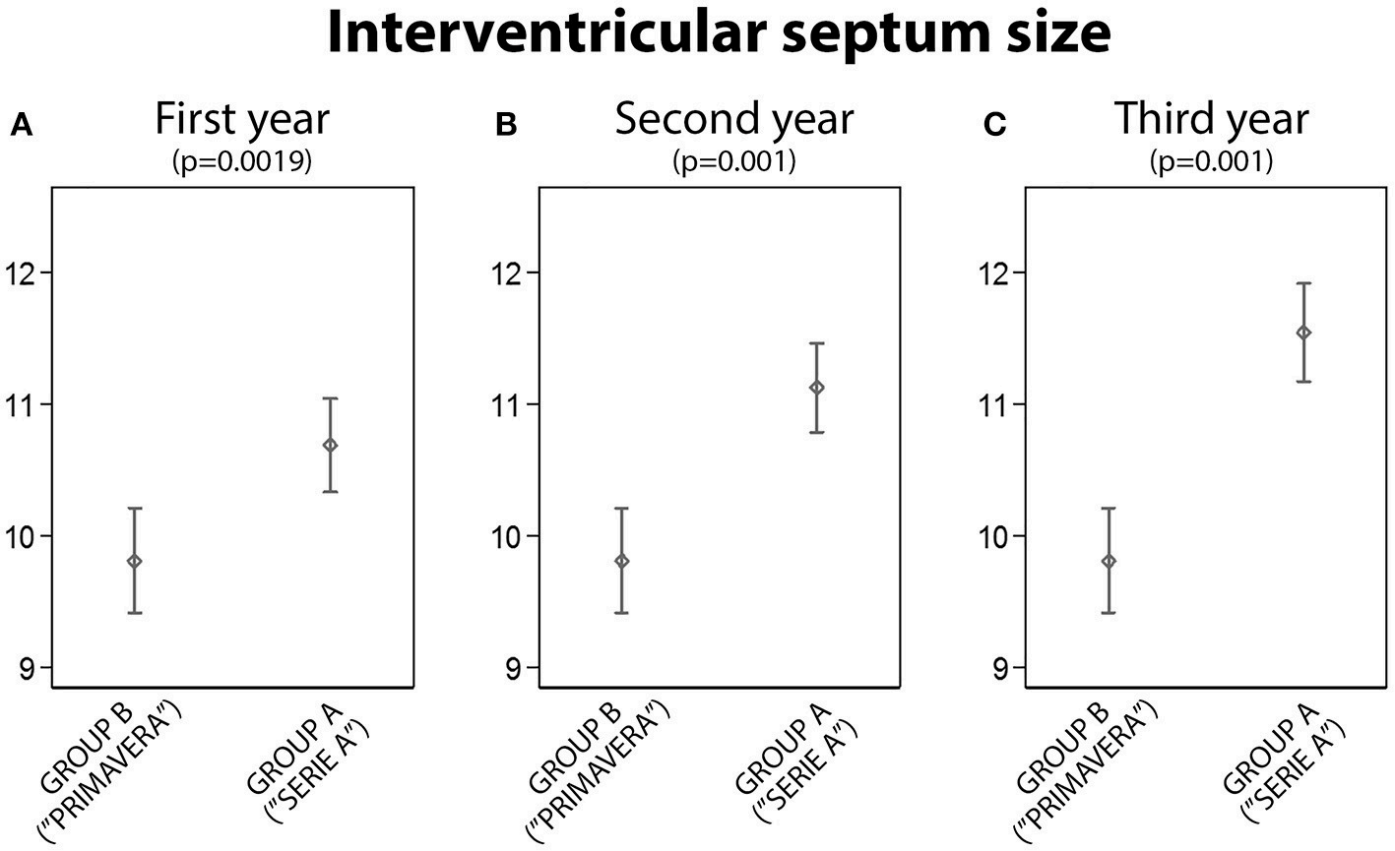
The analysis of intervertricular size between Group A and Group B: statistically significant differences were found in the first, second and third year (**A**, First year; **B**, Second year; **C**, Third year).

The interventricular septum thickness was statistically significant lower in the Group B than in the Group A after the first (*p* = 0.002), second (*p* < 0.001) and third year (*p* < 0.001), Figure 3.

**4. Group A vs. B: Fraction of Ejection Comparison**

**Figure 4:**
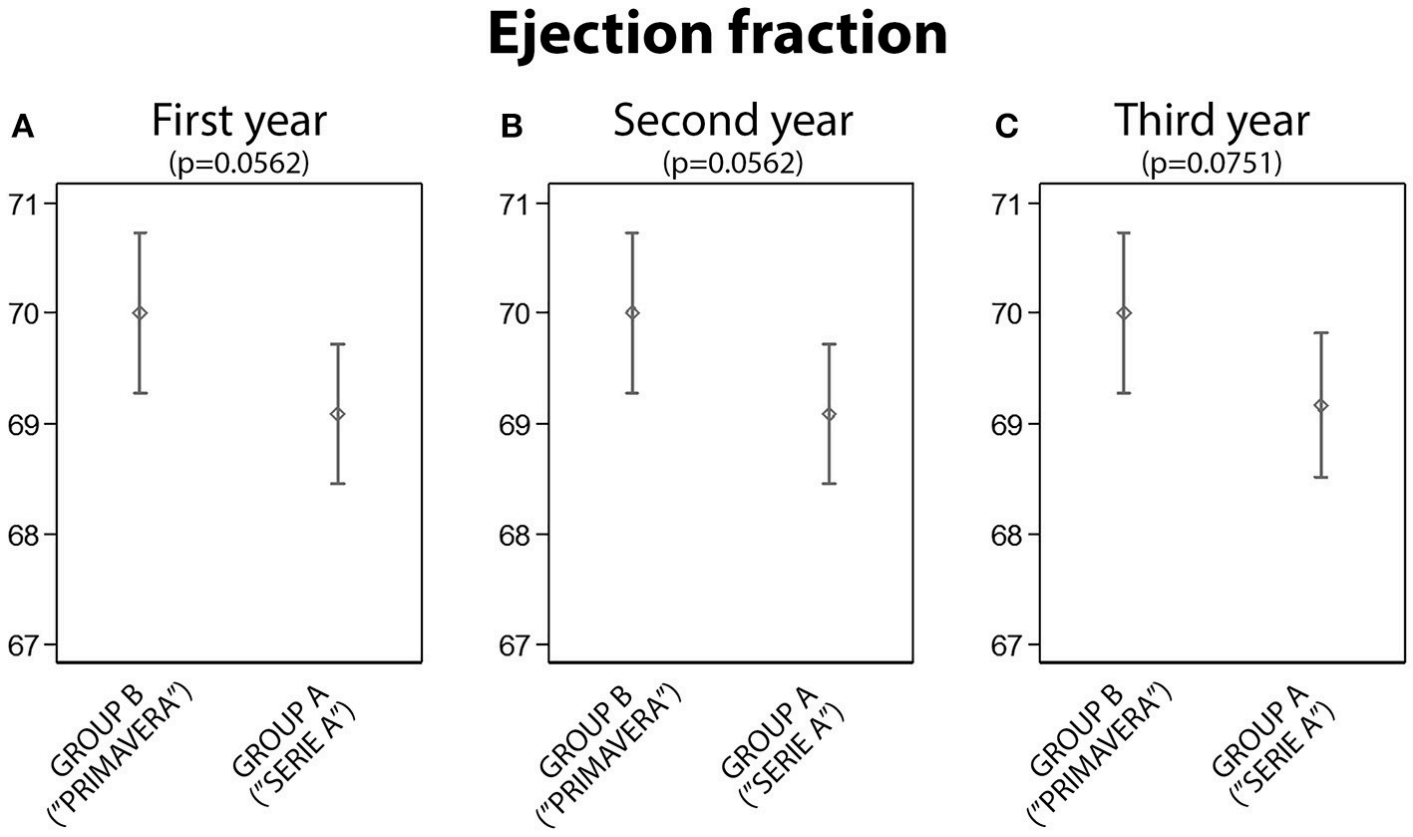
The analysis of the ejection fraction between Group A and Group B: no statistically significant differences were found (**A**, First year; **B**, Second year; **C**, Third year).

There was no statistically significant difference between the A and the B group analyzing the ejection fraction (Figure 4).

**5. Left ventricle size variation during follow-up**

**Figure 5:**
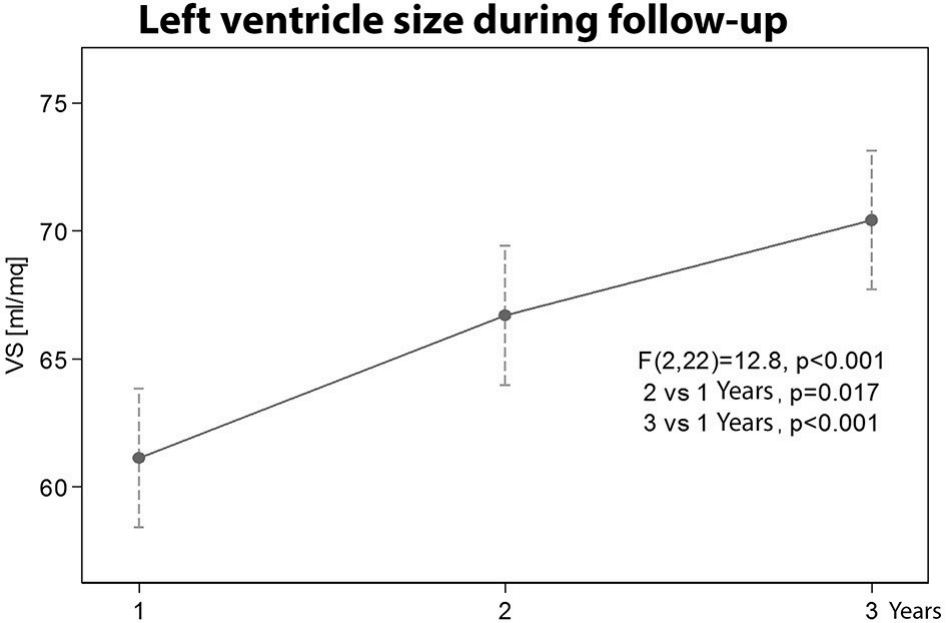
Left ventricle size variation during the three years follow-up.

The left ventricle size of the athletes in the Group A during the 3 years follow-up showed a progressive statistically significant increase (*p* < 0.001). A significant difference was also observed between the first and the second year (*p* = 0.017), and between the first and the third year (*p* < 0.001), as shown in the Figure 5.

**6. Left atrial size variation during follow-up**

**Figure 6:**
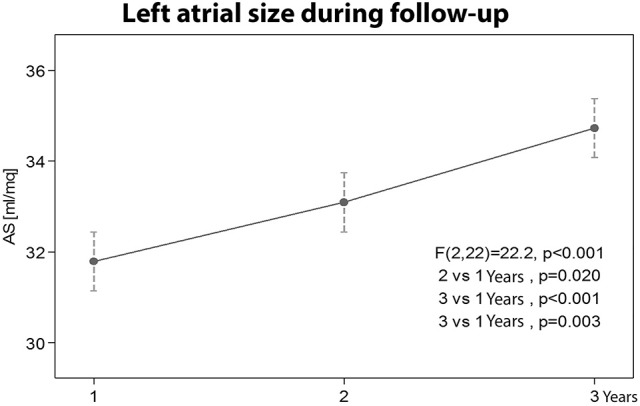
Left atrial size variation during the three years follow-up.

The left atrium size of the athletes in the Group A increased significantly during the 3 years follow up (*p* < 0.001). A statistically significant difference was also found between the first and the second year (*p* = 0.02), between the first and the third year (*p* < 0.001), and between the second and the third year (*p* = 0.003), as shown in the Figure 6.

**7. Interventricular septum size variation during follow-up**

**Figure 7:**
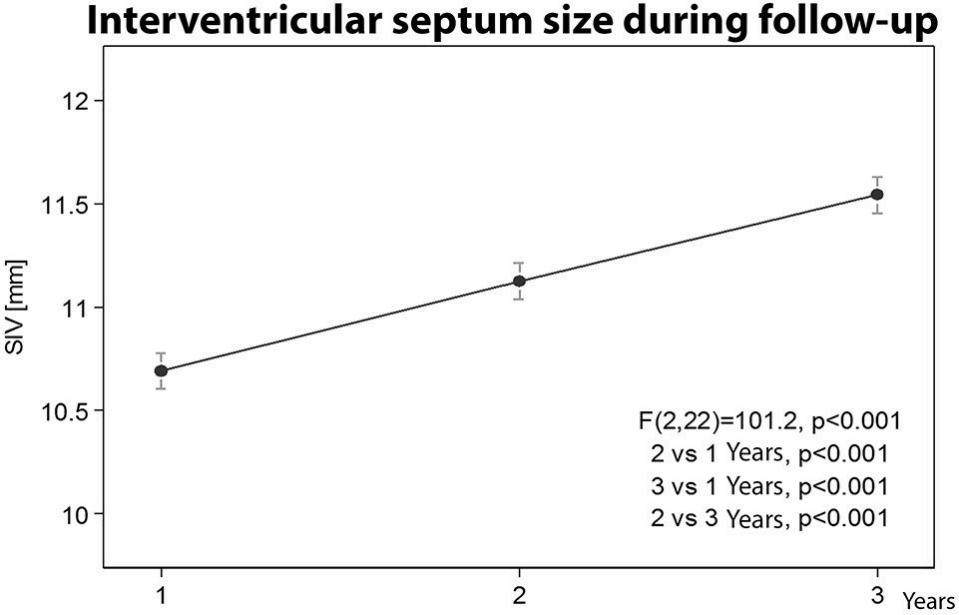
Interventricular septum size variation during the three years follow-up.

The thickness of the interventricular septum in the Group A players showed a progressive statistically significant increase (*p* < 0.001), Figure 7.

**8. Ejection fraction variation during follow up**

**Figure 8:**
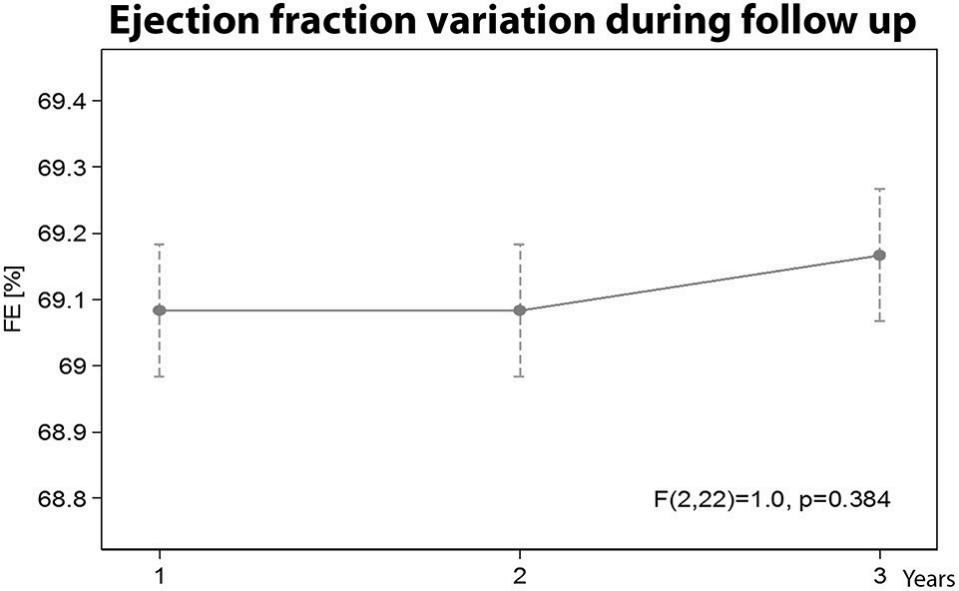
Ejection fraction variation during the three years follow-up.

No statistically significant ejection fraction difference was observed in the Group A athletes during the 3 year follow-up (Figure 8).

## Discussion

Recent guidelines suggested a number of investigations to be performed in order to properly evaluate cardiovascular function in the “Athlete's Heart” (Salomone et al., 2014; Galderisi et al., 2015; Precenzano et al., 2016b).

Athletes should have unaltered diastolic and systolic functions. This is one of the main distinguishing factors from diastolic and systolic pathological degeneration (D'Andrea et al., 2009).

Recent studies showed peculiar echocardiography parameters changes in elite athletes (Vacchiano and Vyshka, 2012; D'Ascenzi et al., 2015).

Several papers focused on athletes' echocardiography features, comparing the study group (athletes) with the control group (sedentary) (Galanti et al., 2016; Precenzano et al., 2016c).

This study focused on two different professional athletes groups: Group A, “Serie A” football team, compared to Group B, composed by the respective B team competing in the “Primavera” league. In both groups, clinical examination and color doppler echocardiography were performed. A number of echocardiography parameters were studied, including: left ventricle size, left atrium size, interventricular septum thickness and ejection fraction. Twelve players of Group A is followed for 3 years, recording echocardiographic parameters.

Athletes of the A group showed a higher left cardiac chambers size and a higher interventricular septum size comparing to the athletes of the B group. No significant differences were found regarding ejection fraction. Such results could be a consequence of a different professional training load.

Following the 12 Group A players, a significant increase in the heart size and interventricular septum size during the second and the third year was recorded, comparing to the data collected from the Group B players.

Such evidence suggests how professional athletes training programs lead to significant heart changes, improving heart function.

According to our data, different training load led to different cardiovascular and echocardiography parameters changes.

During follow-up, no significant differences were found regarding ejection fraction. These data are in accord with previous studies: Bangsbo (1994) reported only small increases in heart adaptations after a 5 week pre-season training period in professional soccer players. Other studies have described that the upper limit to which the ventricle wall thickens with training is 16 mm. These are the maximum heart adaptations and further training cannot influence these parameters (Pelliccia et al., 2010).

Our study confirms that physical activity has significant and relevant effects on the cardiovascular system, leading to structural and functional changes that characterize the “athlete's heart.”

## Conclusions

Our findings confirm the direct physiological effect of physical activity; it can be associated with the “Athlete's heart” changes. All players of both groups were undergone to clinical and instrumental evaluation; it was reported an increase in left cardiac chambers size and interventricular septum thickness. Such changes were much more relevant in long-term professional football players rather than in young football players, even though a considerable increase in ventricular mass and changes in left ventricle strength have been observed in the latter group. The improvement in diastolic function could possibly be due to the increase in ventricular size and performance. Previous studies conducted on diastolic function in the physiologically hypertrophic heart showed maximum increment in left ventricular size, and normal or higher than normal parietal thinning.

Improvements in diastolic function parameters are associated with increased ventricular size and performance. Mitral and tricuspid valves regurgitation are associated with an increase in cardiac chambers size and consequent enlargement of the valve annulus, similar to what occurs in dilated cardiomyopathy. By performing doppler color mapping, we observed that among 45 athletes, 17.6% had mitral insufficiency, 15.5% tricuspid insufficiency, and 11.9% both mitral and tricuspid insufficiencies. It is important to note that in the professional athletes a small amount of doppler regurgitation without any morphological valvular alterations and clinical symptoms, is considerably frequent and should not be considered as a pathological change, since they are due to a “physiological” volumetric increase of the size of the cardiac chambers induced by exercise.

Our work confirmed that professional players who underwent a more durable and intense physical training program had a much higher level of echocardiography parameters than younger professional players. In addition, such data was always below the “Athlete's heart” levels. No significant echocardiographic variation within the examined sample was observed for different roles (goalkeeper, defender, midfielder, or attacker) or skills of individual players.

## Author contributions

Substantial contributions to the conception or design of the work; or the acquisition, analysis, or interpretation of data for the work: CF, FS, MS, IV, GiM, VM, MM, AM. Drafting the work or revising it critically for important intellectual content: CF, FS, LT, GA, FT, MR, GiM, GaM, MPM, GC, AV, CZ, AM. Final approval of the version to be published: CF, FS, GiM, VM, MM, AM. Agreement to be accountable for all aspects of the work in ensuring that questions related to the accuracy or integrity of any part of the work are appropriately investigated and resolved: CF, MS, OC, GiM, AM. All authors read and approved the final manuscript.

### Conflict of interest statement

The authors declare that the research was conducted in the absence of any commercial or financial relationships that could be construed as a potential conflict of interest. The handling Editor declared a shared affiliation, though no other collaboration, with one of the authors OC.
